# ‘No Ski Shoes, Chocolate or Hairsprays’: The (Mis)Adventures of European Companies in China, 1978–88

**DOI:** 10.1177/00220094251323798

**Published:** 2025-03-13

**Authors:** Ariane Knüsel

**Affiliations:** 27211 Université de Fribourg, Département d'Histoire contemporaine, Fribourg, Switzerland

**Keywords:** Sino-European economic relations, China's economic opening, Sino-Swiss joint ventures, Chinese industrial espionage, European companies in China

## Abstract

This article discusses the experiences of Western European companies during China's opening to Western trade and investment in the late 1970s and 1980s. While much has been written about the Chinese policies that led to the introduction of capitalist measures in China, the experiences of European companies have not been covered in detail so far. Using records from several Swiss company archives as well as additional Chinese and Swiss archives and oral history interviews with business representatives and diplomats who were in China during this period, the article analyzes the Swiss experiences in China to discuss how and why European companies tried – and often failed – to take advantage of China's opening, and how European governments and business organizations tried to assist companies in China. It shows how cultural ignorance, international competition, bilateral political and economic relations, the Chinese Communist Party's control over the economy, Chinese industrial espionage and China's lack of knowledge of capitalist terminology and processes affected European attempts to set up shop in China.

At the Swiss Machine Tool Exhibition in Shanghai in March 1979, Deng Xiaoping announced that foreign trade was essential for the People's Republic of China (PRC) to modernize and become stronger. Most Swiss business representatives present interpreted this as the promise of lucrative business deals but eventually discovered that the Chinese wanted to obtain Western technology without having to pay for it.^1^ Johann Jakob Vischer, who was on the pharmaceutical-chemical company Ciba-Geigy's board of directors, was among the few Swiss in Shanghai who remained cautious, advising that the Swiss chemical industry should ‘expect no miracles’ because the situation in China only lent itself to ‘subdued optimism’ and required patience and flexibility.^2^ As this article will show, Western European companies needed more than optimism, patience, and flexibility to break into the Chinese market in the 1980s. Using Swiss and Chinese archival records as well as interviews with business representatives and diplomats who were involved in negotiations in China in the 1970s and 1980s, the following pages will discuss how cultural ignorance and naivety opened up countless pitfalls for Western European companies in China that have so far not been covered by publications on the European presence in China in the 1980s.

Economic reforms had been introduced in China intermittently from 1971 but began in earnest after Mao's death in 1976 and the removal of the Gang of Four, which set the stage for reformers like Hua Guofen, who focused on modernizing China's economy by importing foreign technology without departing from Mao's ideas, and Deng Xiaoping, who championed a move away from self-reliance to a mixed economy.^3^ China's ‘Four Three Programme’ (四三方案) resulted in the large-scale import of equipment and plants from Western countries and Japan but once it became clear that the Chinese lacked the technological know-how to use the equipment and operate the plants, the Third Plenum of the Eleventh Central Committee adopted far-reaching reforms in December 1978, which introduced a market-based economy by liberalizing China's foreign trade and domestic agriculture. Initially, the focus was on reorganizing enterprises and management, as well as on partially opening China to capitalism. Chinese exports were supposed to facilitate the import of new technology and equipment, and the government wanted to encourage foreign investment and technological assistance.^4^

Scholarship on China's turn to capitalism in the late 1970s and 1980s usually focuses on the Chinese government's decisions, measures, economic thought, and the processes, changes, and transformations that the Chinese economy underwent as a result of them.^5^ Some publications discuss Chinese innovation and science and technology measures like the establishment of high-tech parks or talent plans.^6^ Chinese literature on the PRC's economic relations with Europe tends to reflect official interpretations that celebrate spectacular successes and the overcoming of hardships. It does not, however, cover topics like copyright infringement or other illegal ways China obtained foreign technology and know-how.^7^ In the 1980s and 1990s, several publications discussed the efforts of Western companies in China but they primarily relied on laws, official data, and regulations.^8^ More recent publications include archival records, for instance, Martin Albers’ seminal publications on France, Britain and West Germany's efforts in China between 1969 and 1982, and the articles published in a special issue of *Cold War History* in 2017.^9^ Studies of US companies’ experiences in China include Charles Kraus’ fascinating discussion on Coca-Cola in China during the late 1970s and Jim Mann's account of Jeep in China, but European companies have so far been under-researched.^10^ The lack of scholarship can be explained by the fact that company archives are under no obligation to keep the relevant documents, let alone grant historians access to them. This is also a problem because sometimes companies and official publications contradict each other. According to the Chinese authorities, for instance, Edward Keller Ltd. and the China Merchants Steam Navigation Co. Ltd. formed the China Swiss Engineering Ltd., which was officially approved in December 1979 with an investment of USD 286,400 by Edward Keller. Yet, the company (now called Diethelm-Keller), claims that this joint venture had never existed and did not allow me to access company files on negotiations or any other part of business with the Chinese.^11^ This article, therefore, aims to address the gap in the literature by drawing upon records from several company archives, business organization archives and oral history interviews. The following pages analyze the challenges Swiss companies faced in China during the 1980s and compare them to the broader context of Western European business experiences in China during this period.

Economic interests were among the driving forces that propelled the Swiss government to recognize the People's Republic of China on 17 January 1950. The establishment of diplomatic relations between the two countries on 14 September 1950 seemed to position Swiss businesses advantageously in China, even more so once China began utilizing Switzerland as an economic hub in the 1950s and 1960s. Although bilateral trade remained negligible even after the Sino-Swiss Trade Agreement of 1974, Rodolphe Imhoof – counsellor at the Swiss Embassy in Beijing from 1982 to 1984 – felt that Swiss companies played a pivotal role in China's economic opening in the late 1970s and early 1980s due to their advanced technology and Switzerland not being regarded as a geopolitical threat by the Chinese. According to Imhoof, this unique position enabled Swiss businesses to secure favourable deals and negotiate compromises with their Chinese counterparts.^12^

A comparison to other European relations with China, however, paints a different picture. Studies on Sino-Italian relations by Mario Filippo Pini, Enrico Fardella or Guido Samarani and Laura De Giorgi, respectively, as well as Alber's discussion of British, French and West German efforts to promote trade, suggest that the Swiss did not really have a unique advantage over other Western Europeans. Rather than granting genuine preferential treatment to any single nation, China seems to have adeptly orchestrated a complex diplomatic and economic dance, pitting the various Western European countries and their companies against one another. ^13^

In Switzerland, as in the rest of Europe, China's economic reforms sparked great interest. The Swiss Embassy in Beijing even felt compelled to issue a pamphlet on ‘Paths to the Chinese market’. Advising its readers that ‘persistence and patience’ were necessary to be successful in China, it provided information on China's economy and foreign trade, useful contacts in Switzerland, Hong Kong, and the PRC, trade fairs in China, as well as other topics like advertising products in China, organizing symposia and exhibitions in China, inviting Chinese delegations, and sending delegations to China.^14^ The pamphlet was necessary as the Swiss had a long history of stereotypical misconceptions about Chinese business practises that had been shaped by the unequal treaties following the Opium Wars in the nineteenth century and treated the Chinese as a monolithic entity that was unable to appreciate advanced technology (and be willing to pay for it accordingly).^15^

Efforts by the Swiss Embassy in Beijing to dampen Swiss euphoria about the potential of the Chinese market were undermined by the Office Suisse d’Expansion Commerciale (Swiss Office for the Development of Trade, OSEC). OSEC offered extremely popular but very expensive workshops on doing business in China. A one-day workshop, for example, cost CHF 680 but still attracted 400 participants. OSEC also offered annual China business trips, implicitly promising commercial success:

If you want to conquer the ‘bamboo curtain’, it is important to establish direct contact with the Chinese end-user of your product. […] Even an experienced businessman will not find it easy to [establish such contacts.] We can help you.^16^

The trips came with a hefty price tag: a 10-day stay in 1979 cost CHF 5000 per person. Needless to say, many participants were disappointed when they returned empty-handed after being given unrealistic expectations.^17^

The Chinese market turned out to be no smooth sailing for Swiss companies, and Sino-Swiss trade remained negligible until the late 1980s, also when considering Chinese statistics, which include international business by Swiss trading houses (see Figure 1). Even major enterprises like Nestlé struggled in China. Nestlé had spent decades trying to export goods to China, when in 1975 the Chinese finally seemed interested in a collaboration. However, during the subsequent years, Nestlé's representatives realized that China was not so much interested in importing Nestlé products, but rather in setting up a co-packing arrangement in which Chinese luncheon meat, tomato paste, various fruit, mushrooms, and fruit juices were canned according to Nestlé formulas and then exported from China. After three years, negotiations hit a snag when the Chinese rejected Nestlé's demand to inspect the plant while it was running.^18^

**Figure 1.**
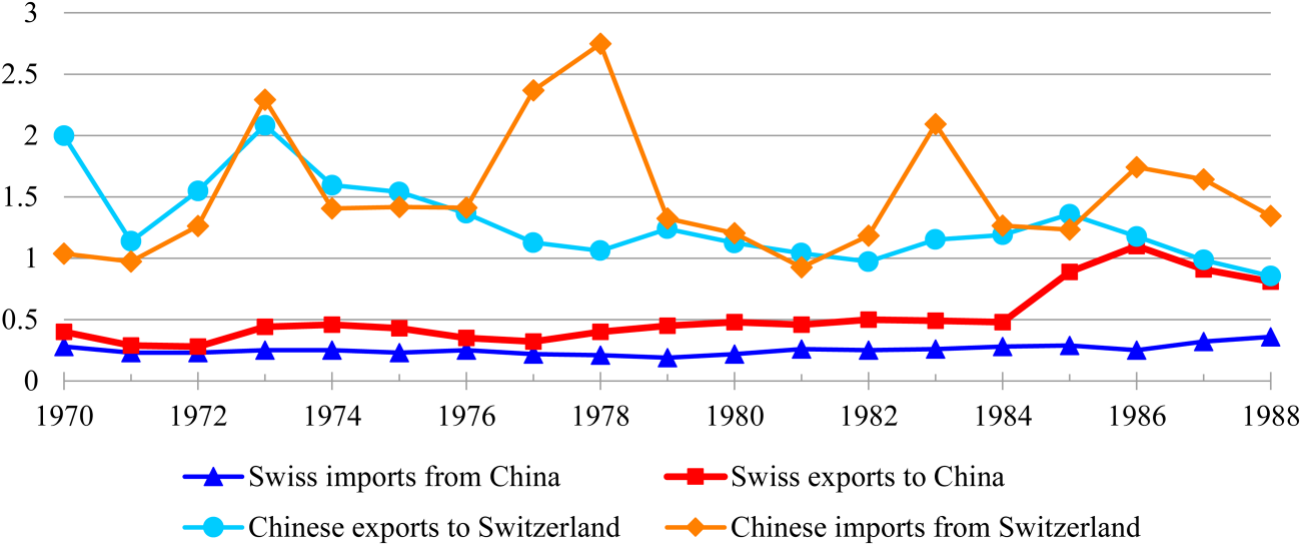
*Sino-Swiss trade in per cent, 1970–1988. Note:* data calculated from Historische Statistik der Schweiz (HSSO), 2012, Tab. L.21, hsso.ch/2012/l/21; HSSO, 2012, Tab. L.25, hsso.ch/2012/l/25; Dangdai Zhongguo congshu bianjipu (ed), *Dangdai Zhongguo duiwai maoyi *(Beijing 1992), 370.

Despite the patchy success of European businesses in China, a 1978 article in the German magazine *Der* *Spiegel* presented Beijing as a consumer's paradise in which ‘shopping is the new magic word’, with stores crammed with customers until closing time, trying to purchase anything from shoes, toys, soaps to tea cups.^19^ Deng Xiaoping's well-publicized charm offensive with Western politicians and numerous visits by economic delegations and politicians further cemented the mirage of a Chinese market with millions of customers waiting to purchase Western goods^20^ and contributed to the competition among Western European countries. Yet, whereas during the historical ‘scramble for China’ in the nineteenth century Western countries tried to carve China into spheres of influence, now China was playing the companies against each other, taking advantage of their ignorance, naivety, and greed.

The ‘gold rush mentality’, as a Swiss official described it, would soon come back to haunt many European companies, which were mesmerized by the potential buying power of the Chinese and underestimated the cultural differences between China and Europe, as well as the vast geographical, economic and cultural differences between different parts of China. Like foreign businesses in previous decades, they underestimated the complexities of operating in the Chinese market. Many fell prey to the ‘dollar-per-person hypothesis’ - the deceptively simple notion that suggests astronomical profits could be achieved if every Chinese consumer purchased a company's product. ^21^ Carolyn Blackman has shown how Western beer producers discovered that the Chinese lacked strong brand loyalty and exhibited seasonal drinking habits, with demand for beer skyrocketing during the summer months and plummeting in Winter due to the Chinese favouring particular drinks for maintaining body heat: ‘The foreign executives discovered that Chinese consumers have their own value and status systems, their own beliefs that affect the way they drink beer.’^22^ Swiss companies encountered similar problems. In 1979, Benedikt von Tscharner, a senior official from the Federal Department of Economic Affairs, reminded an audience of Swiss industrial and commercial representatives that China was not an easily accessible market for Swiss consumer goods such as ‘ski shoes, chocolate or hairsprays’. He advised them to study the Chinese society and economy to formulate a marketing strategy that was suited to the Chinese. Von Tscharner cautioned that selling products in China could take years, that the Chinese were tough negotiators, and that they usually demanded free samples, factory visits, and symposia without guaranteeing a business deal. He concluded: ‘China must definitely not be regarded as a kind of land of milk and honey for Swiss exporters’.^23^

As von Tscharner had foreseen, many Swiss companies encountered issues in China. From 1977, these were discussed in the annual meetings of mixed commissions, which also existed between China and other European countries like West Germany. The Swiss often complained about Chinese demands for lower prices, Chinese copyright violations, and a lack of access to Chinese end-users and to Swiss products in China for Swiss technicians. The Chinese, in turn, criticized the high Swiss prices, Switzerland's prohibition on exporting war material or dual-use items, and references to the Republic of China on Swiss products or brochures.^24^ The discussion about prices in particular highlights the different approaches to production values and technical intricacies between China and Western European countries. According to Rainer Konrad, numerous European companies struggled with quality issues of materials produced in China in the 1980s.^25^ Among the Swiss examples that Rodolphe Imhoof recalled were Chinese manufacturers who prioritized quantity over quality. Insisting that ‘a screw was a screw’, they wanted to produce only one specific type of screw in the highest possible quantity, despite Western companies explaining that screws existed in countless variations and that precise technical specifications were crucial.^26^

In 1978, the introduction of Western joint ventures led to a new era in Sino-European economic relations. In the 1970s, China grappled with ineffective investment and reliance on imports, especially in heavy industry. By 1978, for each CYN 100 that was invested, only CYN 69 of fixed assets and CYN 20 of new national income were added. The solution was to boost domestic production and develop China's heavy industry. In December 1978, it was announced that economic cooperation with other countries would be extended to accelerate China's socialist modernization. Joint ventures were supposed to give China access to Western capital, cutting-edge technology, management skills, and know-how, allowing Chinese enterprises to become international competitors without a net drain on hard currency. Western companies in turn were interested in joint ventures to gain access to China's domestic market and natural resources as well as export opportunities to other Asian markets, and to profit from the sale of technology.^27^

During their visits to Europe in the late 1970s and early 1980s, Chinese leaders and senior officials often mentioned the government's decision to allow joint ventures as a means to boost bilateral trade. According to former Vice Premier Li Lanqing, the idea of joint ventures was first discussed in October 1978 during a negotiation with General Motors. One month later, Deng Xiaoping expressed his approval of joint ventures for car projects.^28^ Swiss archival records corroborate the timeline with such an abrupt policy shift: In September 1978, Chinese officials rejected Swiss proposals for joint ventures, but two months later, Vice-Minister Wang Zhen reversed this stance during a dinner with Swiss officials in Geneva.^29^

Most Western European countries did not establish joint ventures with China until the mid- or late-1980s. Switzerland was an exception, as the Swiss company Schindler participated in China's first industrial Western joint venture. While Li's account only briefly mentions Schindler's joint venture, in the 1980s, numerous publications used Schindler's case to discuss the Chinese measures. The issues encountered by Schindler are also relevant because they are a unique example of early joint ventures.

In the 1970s, China urgently required lift technology for its newly built and planned high-rises, as housing had become so scarce that countless families were forced to live in warehouses, offices, and cellars. Since the elevator industry was not a strategic industry where China could have potentially had its state secrets compromised, it was an ideal way to experiment with Western investment. In December 1978, the Chinese Embassy in Bern arranged for a Chinese delegation to visit Schindler's factory in Ebikon to observe Schindler's elevator technology. Schindler were pleased, for they had wanted to open another production site in Asia to increase their access to Asian markets. The Chinese liked what they saw in Ebikon and invited Schindler to present their technology in China in June 1979 along with a proposal on modernizing China's elevator industry. Schindler and their partner in Asia, Jardine, Matheson & Co. were eventually selected for the joint venture over other elevator companies like Otis Westinghouse and Mitsubishi. According to Uli Sigg, who led Schindler's team in China during the negotiations, the Chinese constantly mentioned Switzerland's early recognition of the PRC in January 1950, and China's good relations with Switzerland, implying that the Chinese were not worried about Swiss espionage. Sigg concluded: ‘It did not hurt to be Swiss’.^30^

Despite Sigg's statement, Switzerland's early recognition of China did not actually lead to widespread preferential treatment of the Swiss in China or more contracts for Swiss companies. In 1979, for example, the Swiss engineering companies Sulzer and BBC were beaten to a large project by a French rival. The Vorort, an organization representing the interests of Swiss industries, had been working closely with the Swiss government in formulating China policies since the 1950s. It noted about the failed deal: ‘It has reaffirmed once again that there might be friendly relations between Switzerland and China, above all because of early diplomatic recognition in 1950, but that these relations do not give us a head start on the economic front’.^31^

A potential stumbling block in negotiations was that Schindler were contractually obliged to insist that Jardine Schindler (Far East) Holdings S.A. were included in the joint venture. After all, Jardine, Matheson & Co. had been involved in the British government's decision to begin the First Opium War and had been the dominant British company in China during the period of unequal treaties. After the Chinese Communists won the Civil War in 1949, they expropriated Jardine's real estate and forced their companies to close. Although Jardine continued to have a terrible reputation in China,^32^ it seems that in 1979, China's need for Western technology overrode anti-imperial sentiment.

On 1 July 1979, the ‘Law of the People's Republic of China on Joint Ventures Using Chinese and Foreign Investment’ officially allowed joint ventures, co-operative exploration agreements, co-production under licence agreements, and compensation trading. It stipulated that foreign investment had to amount to at least 25 per cent of the joint venture's capital, and that ‘technology or equipment contributed by any foreign participant as investment shall be truly advanced and appropriate to China's needs’.^33^ According to Sigg, Schindler only wanted a share of 25 per cent because a larger share was deemed too risky. In March 1980, Schindler Holding AG, Jardine Schindler (Far East) Holdings S.A., and the China Construction Machinery Corporation (CCMC) created the China-Schindler Elevator Co., Ltd. The new company was to produce elevators and escalators primarily for China. Schindler held 15 per cent of the joint venture's equity and Jardine-Schindler ten per cent. The joint venture's paid-in capital amounted to US$16 million, of which US$2.6 million came from Schindler and US$1.4 million from Jardine in cash, and US$12 million from CCMC in the form of buildings, inventory, and machinery in a Shanghai and in a Beijing elevator factory. Sigg also successfully negotiated specific measures for quality control, production levels, and labour productivity. The duration of the joint venture contract was defined as 20 years. China-Schindler was one of the largest joint ventures in China in the early 1980s.^34^

Swiss archival records indicate that in the late 1970s and early 1980s, most joint venture negotiations were either unsuccessful or took several years to reach a deal. In 1978, Ciba-Geigy discussed a joint venture, but a contract was only signed in 1985. The joint venture eventually began operating in 1988.^35^ Nestlé, in turn, spent decades trying to access the Chinese market. An internal document from 1978 expressed considerable frustration with the Chinese insistence on collaborating with Nestlé to export products: ‘Our long-term goal is obviously to open the door to this huge market. At present, the Chinese are not interested in working with us to supply the domestic market, but only to produce for export. This is of less interest to us but, given our long-term goal of ultimately working for the Chinese market, we can accept export cooperation as long as it does not disrupt our business in countries where we already have an industrial presence’.^36^

Ciba's and Nestlé's long-winded negotiations were not unique. During the late 1970s and 1980s, many Western European companies faced significant challenges during negotiations. A major issue was that the Chinese lacked Western legalistic vocabulary due to vast differences in China's legal system. According to Liu Ningshan, from 1949 to 1979, only 68 Western economic treaties were published in China. This limited the spread of Western economic vocabulary in China.^37^ Julian Gewirtz has discussed how China relied on Western economic advisors during China's opening.^38^ However, the measures that were implemented because of these contacts came too late for several negotiations between Chinese and Swiss companies. In 1979, representatives from the Swiss trading house Edward Keller reported: ‘in the conversation one realizes over and over that basic concepts are not understood’ by the Chinese.^39^ Sigg also felt there was no legal common ground during negotiations, as the Chinese had no notion of commercial law, contract law, bond law, company law or tax law. Everything had to be negotiated and defined, including basic concepts like ‘company’, ‘corporation’, ‘governance’, ‘shareholders’, even ‘management’ or ‘board of directors’. As there was no income tax law for joint ventures until September 1980, and a general reform of the profit and taxation system, was only introduced in 1983, Sigg also had to convince the Chinese to define profit and loss accounts as well as profit determination, and how profit was taxed. According to Sigg, Schindler's negotiations had a trailblazing function, as several decisions and concepts were copied by other joint ventures, and some even became part of later legislation, like the tax rate of 33 per cent for profits in mixed companies.^40^

Communication in general was also affected by anti-Western indoctrination. Sigg recalled that China-Schindler's board decisions were discussed endlessly by the Chinese and that Chinese engineers constantly questioned suggestions and technological information. Chinese managers and members of the board of directors who had established mutual trust with Schindler were often replaced by Chinese Communist Party (CCP) officials. The systematic push for anti-Western sentiment seems typical. Rainer Konrad, for instance, has mentioned cases of anti-Western hostility because of weekly Party meetings in joint ventures being mandatory for Chinese employees in the 1980s.^41^

Getting a business deal in China was expensive, as negotiations could easily last up to two years, with foreign business representatives having to travel to China and stay there for several months during this period. They also had to pay for translators and drivers at high prices.^42^ These are examples of what Carolyn Blackman has described as ‘the squeeze’, whereby the Chinese would levy additional expenses on foreigners.^43^ When Rainer Konrad carried out a survey on Western European business with China in the 1980s, one respondent remarked that the Chinese regarded European companies as cash cows to be milked for profit.^44^ For Christoph Blocher, who owned EMS-Chemie, ‘the squeeze’ was not a major issue, just part of doing business in China: ‘You don’t cheat the Chinese. But you have to make sure they don’t cheat you either’.^45^

The Swiss Embassy in Beijing deplored the persistence of the ‘immortal myth of the huge market’ in China among Swiss companies, but there seemed to be no stopping it, particularly as more and more Chinese cities and economic zones adopted preferential policies for foreign investment.^46^ The strong Swiss Franc also increased interest in China's low production costs and the potentially huge market. As a result, companies like Schindler, Sulzer, Landis & Gyr, Sandoz, Ciba-Geigy, and Nestlé, which dominated Swiss investment abroad from 1970 to 2000, were all in China in the 1980s.^47^

In 1979, foreign firms were allowed to have permanent representatives in China. Swiss companies were quick to take advantage of this, particularly once they were allowed to live in several cities. Siber Hegner, for example, installed representatives in Beijing in 1979, in Shanghai in 1981, and in Guangzhou in 1988.^48^ Establishing a permanent office in China was no easy task, as the case of Ciba-Geigy shows: In late 1978 the Chinese authorities asked the Swiss companies Ciba-Geigy, BBC, and Sulzer to open offices in Beijing.^49^ Ciba-Geigy wanted its representative to be a Swiss citizen so that the Swiss Embassy could provide assistance if necessary. Additional requirements included:

Willingness to abstinent lifestyle […] (alcohol, pleasure, etc.).

Sociable despite some isolation.

Calm, patient and always friendly.

Personal interest in the People's Republic of China (people, culture, cuisine etc.).

No susceptibility to political denial or political views.

Good manners and tact.

The person also had to have a high position in the company. Interestingly, Chinese skills were not as necessary as scientific knowledge and excellent knowledge of Ciba-Geigy, including its structure and areas of responsibility. These representatives turned out to be a lucrative source of profit for China. Ciba-Geigy's employee, for instance, moved to the Hotel Xi-Yuan in Beijing, the only available space where foreigners could rent offices. However, the rent was exorbitant, and by 1985 cost CHF 120 per day despite the small rooms, so Ciba-Geigy relocated its office to one of the newly built high-rises for foreign offices.^50^

Europeans in China quickly encountered corruption. Most of the business representatives and diplomats interviewed for this article mentioned how endemic corruption was in China in the 1980s. While Christoph Blocher felt that the entire system was based on corruption at that time, Rodolphe Imhoof recalled that local and provincial officials tended to be particularly corrupt.^51^ Blackman explains the pervasiveness of corruption with officials interpreting laws and regulations in their jurisdiction, including ‘the issuing of permits for construction, power, water and other facilities’.^52^

Corruption in China was also influenced by Confucianism's definition of the individual according to its relations with and behaviour towards the family and Chinese society in general. Successful business representatives had to have ‘guanxi’ (关系 = relations with relevant people that are based on loyalty and mutual obligations) and adhere to the principles of reciprocity and sharing.^53^ Western ignorance or even violation of these principles could affect the course of negotiations. Lavish banquets with toasts and entertainment were usually part of the process of negotiation and building these ‘friendships’ but some Swiss struggled with Chinese cuisine. Blocher recalled that he would always make a point at official dinners to try every dish and laud it, even when the rest of his family turned away in disgust.^54^

The necessity of knowing the right people was also expensive. As more and more Chinese provinces, cities, as well as industrial and commercial departments set up import and export trading companies in the 1980s, European companies were forced to send more representatives to China if they wanted to do business in several Chinese regions. The trading house Siber Hegner was able to send staff to several provinces to negotiate contracts with local corporations. Their experience and contact network gave them an edge over fellow competitors, and by 1987, most of the CHF 300 million worth of silk exported from China by Swiss trading houses went to other countries, not Switzerland. However, smaller trading houses did not have the means to fund the presence of several representatives in China, and consequently, they lost contracts.^55^

Relations with relevant trade representatives were also forged in Europe. European companies spent an enormous amount of money on wining and dining visiting Chinese delegations in the 1980s. In 1980 alone, 60 Chinese delegations visited Switzerland.^56^ According to Frank Bösch, some Chinese delegations visited West Germany for over a month at a time, during which time they were hosted by German organizations.^57^ Blocher built strong relationships with CCP provincial officials by inviting them to EMS-Chemie's headquarters in Switzerland and hosting them at Castle Rhäzüns, where he showcased a Swiss music box that had once belonged to a Chinese emperor. He is convinced that these visits significantly improved relationships with party officials.^58^

Among the companies struggling with difficult and expensive negotiations, was Sulzer, which declared in 1980: ‘it is a waste of time and money if every Swiss firm makes the same experiences that others have previously made’.^59^ As a result, in September 1980, the Sino-Swiss Chamber of Commerce (SCCC) was founded with Uli Sigg as President for the first 12 years. It facilitated contact with Chinese officials and business representatives and offered assistance and information on products, statistical data, relevant authorities and companies, laws and regulations, etc. By 1982, the SCCC had over 150 members.^60^

It is unclear how many European joint ventures were actually set up in China. Jiang Yu and Zhang Xiaozhong claim that by 1984, 931 joint ventures had been established, but according to Liu, there were only 93 by 1983. ^61^ There are no official numbers for Swiss joint ventures, as companies were not obliged to notify the government about them. By 1989, the Swiss Embassy in Beijing knew only of 17 Sino-Swiss joint ventures, including Mövenpick Radisson hotels, Nestlé milk products, F. Hoffmann-La Roche vitamins, and Feldschlösschen alcohol-free beer.^62^ Considering that dozens of projects had failed, it is, perhaps, more fitting to refer to ‘joint misadventures’ as opposed to ‘joint ventures’.

Why did so many joint venture negotiations fail? Archival records dealing with the failed efforts of Western European companies in China are difficult to access, maybe because it could damage the reputation of the companies. Nevertheless, there are some possible explanations. Firstly, the Chinese seem to have been indiscriminate in negotiating with European companies, as local and provincial authorities tried to set up joint ventures. In December 1978, for example, the Chinese were discussing four different joint ventures with Edward Keller.^63^ This scattergun approach seems to have been made with the expectation of a high fail-rate. Secondly, the Chinese demands may have been too harsh for many European companies, and theyfocused on doing business with the USA and European countries instead.

A third explanation is connected to the lack of connections in China. Albers claimed that West Germany had an advantage over other Western European countries in China because it could rely on China experts to engage in economic diplomacy. In a country like China, where special attention is placed on formalized behaviour, the decades of experience of men like Otto Wolff or Ambassador Ernst Wickert were of tremendous importance.^64^ Switzerland had a much more critical view of such people. During the Cold War, the Swiss Federal Police suspected anyone who engaged in business with Eastern bloc nations of being involved in criminal activities like embargo goods dealing or espionage. France and Britain seemed to have similarly questioned the communist leanings of some of their economic intermediaries.^65^ In any case, by the 1970s, Switzerland lacked commercial intermediaries who were as well-connected in Chinese business circles as their German counterparts, and companies had to rely on Swiss diplomats in Beijing as well as Swiss organizations like OSEC, whose questionable approach has already been discussed.

Cultural differences seem to have been another major obstacle. Some European business representatives discovered that the Chinese had a different set of ethically acceptable business practices. Caroline Blackman has argued that the ‘free-for-all regulatory environment and an immense desire to get rich following the commercial repression of the communist period’ led to what was perceived in the West as ‘unscrupulous and unethical practice’. If we add China's traditional lack of ‘strong commercial legal infrastructure to support good practice or to penalize defaulters’,^66^ it seems logical that many European companies were taken advantage of by their Chinese (potential) partners. In 1980, the Swiss Embassy warned overeager companies in Switzerland: ‘Sensational profits from joint ventures in China are […] not to be expected. The Chinese market is certainly no Eldorado’.^67^

Of course, not all European companies in China tried to set up joint ventures. Schindler's case had demonstrated that joint ventures in China were not a walk in the park but required seemingly endless negotiation, patience, and sensitivity to cultural differences. A considerable number of Swiss companies instead chose to enter licencing deals and cooperation agreements. Sulzer on principle preferred licensing deals in Asia, while Brown Boveri relied on licensing deals with China until the mid-1980s because they found joint ventures with China too risky. Ems-Chemie, in turn, sold China outdated technology it could not sell in the West anymore.^68^ By 1989, approximately 30 Swiss companies had entered co-production, cooperation or licensing deals with Chinese companies. West Germany also had more cooperation and licensing deals than joint ventures, at least until the mid-1980s.^69^

In the 1980s, Swiss companies lost many contracts to other Western companies. The Chinese usually blamed the high Swiss prices for this, but they also favoured business deals with countries whose political clout was stronger than that of Switzerland.^70^ While other European countries were keen to negotiate treaties and agreements on the protection of investment and on double taxation, Switzerland only did so from 1985 onwards.^71^ Favourable interest rates, for instance, allowed Sulzer's French subsidiary CCM to sell a diesel engine to China at a price 25 per cent lower than Sulzer's, despite having production costs.^72^

Italy concluded a cooperation agreement with China that used USD 48 million in credit and USD 24 million in grants from Italy's development cooperation programme (cooperazione allo sviluppo) from 1982 to 1984 and was tied to Italian projects and exports. As a result, loans could be offered on more favourable terms than typical commercial credits, and investment doubled from 1984 to 1985. A second agreement for USD 576 million in credit and 95 million in grants ran from 1987 to 1989. These agreements gave Italian companies the advantage of offering better terms than other Western European companies in China, and they managed to increase exports to China in the mid-1980s more than any other Western country.^73^ Switzerland's first mixed loan, in contrast, ran from 1985 and consisted of CHF 80 million to China in 1984. It was tied to Swiss exports and was eventually used for 23 projects. Another mixed loan was signed in 1987 for CHF 100 million.^74^ In the same year, BBC and Sulzer concluded a deal for turbines, generators, and other machinery for a coal-fired power station for CHF 200 million. The deal was financed by a loan of CHF 155 million by a Swiss banking consortium to the Bank of China, the first time Swiss banks managed to conclude a loan agreement with China on a private basis.^75^ These examples show that the Chinese very successfully played not only European companies but also governments against each other, exploiting their fascination with the potential profits of the Chinese market.

Not everybody welcomed business deals with China. By the 1980s, Swiss silk textile producers suffered from the import of cheap Chinese textiles, particularly silk scarves, ties, and shirts. Chinese silk textiles exports to Europe rose from 121,000 kg in 1985 to 1,100,000 kg in 1989, devastating Switzerland's export-oriented silk industry. Chinese silk textile exports to Switzerland more than tripled. Silk shirts produced in Switzerland cost CHF 300 and could not compete with Chinese ones sold for CHF 30. Dumping prices and oversupply also affected the reputation of silk as a luxury material, causing customers to purchase pashmina or cashmere products instead.^76^ However, some Swiss companies profited from the Chinese textile industry's boom. Ems-Chemie sold synthetic fibres to China, for which it was castigated by the Swiss Textile industry, and when Sulzer entered an agreement to export looms to China in return for worsted fabric in 1985, Swiss tweed producers threatened to inform the public about Sulzer's actions.^77^

China's reforms also affected management because planned economy had led to an imbalance of supply and demand, low product quality, and a disregard for profit and loss. When 260,000 plants and factories were decentralized in 1970, management descended into utter chaos. Miscommunication and conflicting directives between local and central authorities severely impacted industrial efficiency and productivity. After various measures failed to improve the issues, it was decided in December 1978, that local authorities and enterprises had to be granted greater autonomy in the operation of enterprises and their production.^78^ China-Schindler was among the pioneering joint ventures that helped reform China's management system and communication. China-Schindler's elevator factories in Shanghai and Beijing improved communication between the different departments and introduced a monitoring system for managerial instructions and quality control. However, the changes were slow to take effect. In 1987, Sigg still bemoaned the Chinese employees’ unwillingness to take responsibility and delegate decisions.^79^

Among the skills that the Chinese managed to learn from Western companies was marketing. Until the 1980s, the state had determined what was to be produced in China, in which quantity, at what price, and for whom, turning advertising into a ‘capitalistic evil’.^80^ Before China-Schindler, lots were drawn to distribute elevators, which were assembled and installed by local companies. The elevator company in Shanghai only employed one part-time salesman. Schindler-China, by contrast, opened a showroom and sales offices in several Chinese cities. Other Swiss companies also brought marketing strategies with them. When Ciba-Geigy established a joint venture in China, they relied on marketing strategies from Ciba-Geigy's Hong Kong team.^81^

A major issue for all companies trying to do business in China was copyright infringement. During joint venture negotiations, the Chinese usually demanded the most advanced technology. Often, this was not possible because of a lack of suitable equipment or skilled workers. Thus, China-Schindler's subcontractors in China lacked the technology to produce some parts. There were also linguistic issues: When Schindler handed the Chinese 500 ‘binders of documents and thousands of blueprints’ in French and German the Chinese had problems translating them.^82^ Other companies outright refused to share innovative technology out of fear of copyright violations, which were rampant in China. Sigg recalled that when it came to patents and usage licences in China, it was like the ‘Wild West’. Schindler-China had over 100 Chinese subcontractors that produced thousands of components for which they received detailed instructions from Schindler. Many subcontractors ended up illegally selling these parts to competitors around the world, some very brazen ones even referred to them as ‘Schindler technology’.^83^

Schindler's issues were typical for European companies relying on subcontractors in China. Since its founding in 1949, the PRC has relied on espionage to increase industrial and agricultural productivity and close the scientific and technological gap with the West. As access to relevant Chinese and European (counter)intelligence records is severely restricted, there are relatively few academic publications on Chinese industrial espionage in Europe, and they focus either on the period after 1990 or on the 1950s and 1960s.^84^ However, the 1980s marked a transformation in Chinese industrial espionage as China's opening gave the Chinese government new opportunities to access Western technology, research, and development.

From the 1950s to the 1970s, Chinese industrial espionage in Europe was orchestrated from the Chinese Trade Office in Muri, Switzerland, and focused on obtaining technological information that allowed China to copy production processes and products, or on embargoed goods such as weapons or material required for the production nuclear bombs. The staff of the Trade Office in Muri or visiting trade delegations inspected Swiss companies and factories on an almost weekly basis. Intelligence officers would try to use these factory visits to gain access to relevant science, technology, and production processes. This approach continued in the 1980s, with the Chinese using trade delegations to study Swiss production processes to improve the quality of Chinese products and make them more marketable.^85^ As a result, companies like BBC ordered their employees to avoid any communication with the Chinese Embassy unless it had been discussed with their superiors.^86^ Not all companies were alarmed by Chinese industrial espionage. Ems-Chemie had cases of Chinese patent violations and illegal photographing but the company tried to avoid this by prohibiting Chinese interns from entering certain areas.^87^

Another traditional method of industrial espionage included inviting European companies to exhibit their products in China or asking them for presentations on their products and then systematically mining them for relevant information on the technology and production processes.^88^ During China's opening, business negotiations continued to be used by the Chinese to obtain know-how and technology without buying products. In 1980, the China New-Type Buiding Materials Company demanded that the Swiss shoe producer Bally hand over ‘as many documents as possible’ about Bally's technology, claiming that this was necessary to decide whether or not a Bally delegation would be invited to China to discuss a potential collaboration.^89^ China's exploitation of preliminary discussions and visits for knowledge transfer without concluding contracts for specific services later on was so rampant that some companies organized workshops for their Swiss employees on marketing, negotiating, and know-how transfer in China.^90^

One way in which the 1980s were a pivotal moment in Chinese industrial espionage was because joint ventures often required Chinese employees to learn necessary skills in Europe, where they were trained for several weeks or months. In 1981, for instance, 12 Chinese workers arrived at Schindler Works in Ebikon in 1981 where they were trained for four months. Practical training like this was often demanded by the Chinese during the negotiations but not every company gave in to the Chinese demands. In the early 1980s, the Chinese Embassy in Bern repeatedly tried to place interns with the BBC, particularly in plasma physics and nuclear automation. However, for the company, this was out of the question. ^91^

In the 1980s, the presence of European companies’ joint ventures, factories, and showrooms in China made them even more vulnerable to industrial espionage, as the case of wristwatches shows. Wristwatches had long been popular in China but, as Karl Gerth has discussed, China had lacked the technology to produce them in a quality that rivalled Western or Japanese watches. Swiss watches in particular were a status object par excellence in communist China, with about 80 per cent of imported watches coming from Switzerland. Whenever Chinese officials travelled to Switzerland, they would purchase Swiss watches for themselves or their family members. Even Zhou Enlai purchased a watch for his wife in Geneva in 1954. Regular members of Chinese society had to make do with domestically produced watches that relied on Soviet and Japanese parts as well as Soviet and Swiss equipment. By 1978, more than 13 million watches were produced annually.^92^ It is telling that China's first foreign TV commercial was for a Swiss Rado watch, airing in 1979.^93^

In the 1950s and 1960s, the Chinese illegally imported watchmaking machines and apparatus from Switzerland and demanded that Swiss watch exhibitions in China include individual watch parts and watchmaking machinery, but they never managed to produce watches that rivalled the quality of authentic Swiss watches. ^94^ The 1980s, however, were a game-changer for Chinese industrial espionage in the watchmaking industry because Swiss watchmaking companies set up shop in China. In 1980, Longines and Omega opened watch centres in Shanghai,^95^ while in 1981 a Swiss watch centre opened in Beijing with three floors of sales and reparations and 70 Chinese employees. One Swiss company alleged that it had been forced to close its Chinese factory due to constant staff poaching, with Chinese authorities systematically using it to train Chinese watchmakers, causing such a high staff turnover that the Swiss barely managed to train Chinese employees before the authorities made them leave and work for a Chinese watchmaker. The authorities also continually demanded that the employees’ wages be increased by 10 per cent until the factory was no longer deemed profitable, causing its general director to accuse China of using the factory as an ‘educational workshop for China's watchmaking industry’.^96^ In the late 1980s, Rainer Konrad found that high staff turnover in joint ventures was a particular problem for Western hotel chains in China, but Swiss archival records and media reports, as well as a publication by Yingyang Wang suggest that it was actually a common issue.^97^ As China obtained Western technology, it also became more adept at copying European products. The Swiss silk printing industry, for instance, suffered because new printing techniques allowed Chinese factories in the 1980s to illegally copy Swiss designs and print them only a few weeks after the Swiss silk producers had presented their designs.^98^

Among the Swiss companies that became victims of Chinese copyright violations were several pharmaceutical and chemical companies, including Ciba-Geigy. In 1981, the company's representative in Beijing discovered that the Chinese had illegally copied Ciba-Geigy's two most profitable products, Tegretol and Regitin, as well as some products that the company was planning to introduce to China. For Ciba-Geigy, this was a financial disaster. The entire process of developing a drug was estimated to take about 20 years and cost approximately CHF 100 million. Swiss chemical-pharmaceutical companies were so frequently targeted by Chinese copyright infringements that in 1987 the Swiss Society of Chemical Industries prioritized a revision of Chinese patent laws for the mixed commission.^99^

It seems that no Western product was immune to Chinese counterfeiting. Even candy was reproduced without a license. M&Ms, for example, were copied illegally and sold in almost identical packaging soon after they were marketed in China. The same thing happened with Sugus, a Swiss candy produced by Jacobs Suchard, S.A. In 1974, no Chinese buyer was willing to import Sugus to China. However, in the following years, several cases of pirated Sugus were discovered in China, one even called the sweets ‘sugus’. One case was discovered when a Swiss commercial delegation spotted the counterfeit sweets during a factory visit. In another case, fake Sugus were distributed on an Air China flight. A particularly brazen case of fake Sugus production was carried out by the Chinese Guangzhou National Phoenix Candy Factory. Their sweets were not only wrapped in paper labelled ‘SUGUS’ and put in a bag that was designed like the Swiss Sugus bag, but they were also named *Rui Shi Tang* (瑞士糖 Swiss sweets). How exactly the Chinese managed to produce sweets that looked and tasted like the Swiss Sugus remains unknown. It was quite a feat, however, because Suchard had shrouded their recipe in secrecy, only sharing the candy paste with licensees. ^100^

Western foodstuffs seem to have been liable to piracy. Charles Kraus has shown that after the Cultural Revolution's demonization of Western food and drink, Chinese efforts to obtain the equipment and copy the manufacturing techniques of famous Western food and beverage makers increased. The influx of foreign tourists also meant that money could be made by selling products that appealed to their tastes. While Kraus mentions that Western beverages were imported to be sold to tourists,^101^ the case of the counterfeited Sugus distributed on flights seems to hint at Chinese efforts to corner at least part of that particular domestic market with counterfeit Western goods.

The preceding pages have shown that the opening of China in the 1970s and 1980s marked a new chapter in Sino-European commercial relations. Although Schindler was a trailblazer as the first Western industrial joint venture in China, the typical experiences of Swiss companies in China seem to mirror those of other Western European companies. Access to the Chinese market took months if not years and a lot of money, and it was not guaranteed. Many European products did not suit the Chinese market. Some companies discovered that the Chinese had engaged in industrial espionage or used the negotiation process to access the required know-how and technology. The Chinese negotiators’ lack of knowledge of Western terminology made negotiations difficult, but even once deals had been reached, China's seemingly complete disregard for patents and copyrights forced European companies to formulate long term goals instead of expecting quick profits.

Western European companies were not simply taken advantage of by the cunning Chinese Communists, but the Chinese seem to have mastered the art of playing European companies against each other. This strategy was also successful because many companies were driven by a mixture of (cultural) ignorance, naivety, and greed. Although China's policies as well as bilateral diplomatic and economic relations influenced a company's chances of setting up shop in China, many companies lacked an understanding of Chinese culture and the institutional and political intricacies. Whether this was more common in Switzerland than in the rest of Europe requires further research.

The presence of Chinese in Western Europe as a result of joint ventures and other collaborations deserves to be analyzed in more detail. This includes the presence of Chinese spouses that Europeans met while being posted in China as well as the Chinese interns who were trained in European towns and cities. The presence of European companies in China and the use of Chinese interns in Europe also opened up new ways for China to engage in industrial espionage. More research on the individual companies’ experiences is needed to trace patterns, success rates, and pervasiveness of China's industrial espionage in Europe in the late 1970s and 1980s. This topic is particularly important because Chinese industrial espionage remains a problem for European companies.

## Notes

1 ‘China – Vorbereitung der agrartechnischen Ausstellung in Peking – 1978’, 19 July 1978, Archiv für Zeitgeschichte (AfZ), IBVA 380.3.9; ‘Ruishi jichuang zhanlan hui yijiuqijiu nian san yue shisi ri zhi ershiwu ri ’, undated, Shanghai Municipal Archive (SMA) H1-26-13; Shi die er Shangye Ju Geming Weiyuanhui to Yan Youmin , 24 March 1979, SMA B98-6-16-8.

2 Vischer to Egli, 27 March 1979, Novartis Archiv **(**NOA) FCG RD 9.1.10.

3 O. A. Westad and C. Jian, *The Great Transformation. China's Road from Revolution to Reform* (New Haven and London 2024); P. Millwood, ‘China's Abandonment of Self-Reliance and Sino-American Opening before 1979’, *Cold War History*, (2024), 1–22, https://doi.org/10.1080/14682745.2024.2355191; E. F. Vogel, *Deng Xiaoping and the transformation of China* (Cambridge 2011); F. C. Teiwes and W. Sun, ‘China's New Economic Policy under Hua Guofeng: Party Consensus and Party Myths’, *The China Journal*, 66 (2011), 1–23.

4 L. Liu, ‘China's Large-scale Importation of Western Technology and the U.S. Response, 1972–1976’, *Diplomatic History*, 45, 4 (2021), 794–80; F. Bösch, *Zeitenwende 1979: Als die Welt von heute begann* (2nd edn, München 2019), 141–186; Jiang Y. and Zhang X. (eds), *Zhongguo Jingji Fazhan Shi (1949–2005) *, vol. 1 (Shanghai 2007)*,* 673–6 and 700–1; Vogel, *Deng Xiaoping*, 229-48; M. Taube, ‘Economic Relations between the PRC and the States of Europe’, *The China Quarterly*, 169 (2002), 78–107*.*

5 S. Li, *The Rise of China, Inc.: How the Chinese Communist Party Transformed China into a Giant* Corporation (Cambridge 2022); J. Gewirtz, *Unlikely Partners: Chinese Reformers, Western Economists, and the Making of Global China* (Cambridge 2017); M. Yu, ‘China's international trade development and opening-up policy design over the past four decades’, *China Economic Journal*, 11, 3 (2018), 301-18; Li L., *Breaking Through: The Birth of China's Opening-Up Policy* (Oxford 2009); V. Mantzopoulos and R. Shen, *The Political Economy of China's Systemic Transformation* (New York 2011); N. R. Lardy, *Foreign Trade and Economic Reform in China, 1978–1990* (Cambridge 1992).

6 L. Miao, J. Zheng, J. A. Jean, Y. Lu, ‘China's international Talent Policy (ITP): The Changes and Driving Forces, 1978–2020’, Journal of Contemporary China, 31,136, 644-61; Y. Sun and C. Cao, *The Political Economy of Science, Technology and Innovation in China: Policymaking, Funding, Talent, and Organization* (Cambridge 2024); R.P. Applebaum, C. Cao, X. Han, R. Parker, D. Simon, *Innovation in China: Challenging the Global science and Technology System* (Cambridge 2018).

7 Jiang and Zhang, *Zhongguo*; Zheng Y. (ed), *Zhonghua Renmin Gongheguo jingji shi (1949–2019) *(2nd edn, Beijing 2019); Liu N., *Xin Zhongguo Minying jingji sixiang yanjiu, 1949–2019 *(Beijing 2019); Pei C. and Liu B., ‘The Economics of China's Opening Up: Developing an Economic Theory That Explains China's Achievement’, *Social Sciences in China* 42, 1 (2021), 53–76; Li L., *Breaking Through: The Birth of China's Opening-Up Policy*, trans. Ling Y. and Zhang S. (Oxford 2009) 241-7.

8 Taube, ‘Economic Relations’; S. P. S. Ho and R. W. Huenemann, *China's Open Door Policy: The Quest for Foreign Technology and Capital* (Vancouver 1984); R. Pomfret, ‘Ten Years of Direct Foreign Investment in China’, *Asian Perspective,* 13, 12 (1989), 35–53; M. M. Pearson, *Joint Ventures in the People's Republic of China: The Control of Foreign Direct Investment under Socialism* (Princeton 1991).

9 M. Albers, Britain, France, West Germany and the People's Republic of China, 1969–1982 (London 2016); M. Albers, ‘Partners but not Allies: West European Co-operation with China, 1978–1982’, *Diplomacy & Statecraft*, 25, 4 (2014), 688–707; Special Issue *Socialism, Capitalism and Sino-European Relations in the Deng Xiaoping Era, 1978–1992*, *Cold War History*, 17, 2 (2017).

10 C. Kraus, ‘More than Just a Soft Drink: Coca-Cola and China's Early Reform and Opening’, *Diplomatic History* 43, 1 (2019), 107–29; J. Mann, *Beijing Jeep: A Case Study of Western Business in China* (New York 2018 [1997]); M. Posh, *1000 Tage in Shanghai: Die abenteuerliche Gründung der ersten chinesisch-deutschen Automobilfabrik* (München 2006).

11 ‘A list of joint ventures using Chinese and Foreign Investment’, in Policy Research Department and Foreign Investment Administration, Ministry of Foreign Economic Relations and Trade (ed), *Guide to China's Foreign Economic Relations and Trade: Investment Special, Hong Kong: Economic Information and Agency* (2nd edn, Hong Kong 1984), 311–2; Diethelm-Keller (C. Alborghetti), correspondence with A. Knüsel, 5 February 2019.

12 R. Imhoof, interview with A. Knüsel, 26 January 2018.

13 M. F. Pini, *Italia e Cina: 60 anni tra passato e future* (Roma 2011*),* 168–79; G. Samarani and L. De Giorgi, *Lontane, vicine: Le relazioni fra Cina e Italia nel Novecento* (Roma 2015 [2011]), 137–43; E. Fardella, ‘ A significant periphery of the Cold War: Italy-China bilateral relations, 1949–1989’, *Cold War History*, 17, 2 (2017), 193.

14 Swiss Embassy in Beijing, ‘Wege zum chinesischen Markt’, March 1978, AfZ IBVA 1978 380.3.8; Swiss Embassy in Beijing, ‘Wege zum chinesischen Markt’, November 1978, AfZ IBVA 1978 380.3.8.

15 A. Knüsel, ‘A Tricky Business: Swiss Perceptions of Informal Imperialism in China in the 1920s’, *Journal of Modern Chinese History*, 2 (2014), 210–29.

16 Piotet and Kellerer, ‘Volksrepublik China’, 7 March 1985, Swiss Federal Archives (SFA) E7115A#1993/340#1905*.

17 OSEC, ‘Business Guide Volksrepublik China’, 1 January 1980, AfZ IBVA 380.3.12; Ducrey to Schweizerische Zentrale für Handelsförderung, 21.1.1980, AfZ IBVA 380.3.12; Müller to Bettschart, von Tscharner and Clerc, 20 June 1979, Swiss Federal Archives (SFA) E7115A#1990/60#2227*.

18 Muggli, ‘Visit to China (Canton + Peking)’, 9 May 1978, Nestlé Archive (NA) CH AHN NES C.1.5./4250.

19 ‘Einkaufen heißt das neue Zauberwort’, *Spiegel*, 28 May 1978, https://www.spiegel.de/politik/einkaufen-heisst-das-neue-zauberwort-a-37176d41-0002-0001-0000-000040617911 (accessed on 30 December 2024).

20 Liu, ‘China's Large-scale Importation’, 794-80; Bösch, *Zeitenwende*, 141-86.

21 Atlas-Service AG, ‘Realitäten und Illusionen im China-Handel’, 27 June 1979, AfZ IBVA 380.3.7 (quote); Imhoof, interview; L. S. T. Tai, ‘Doing Business in the People's Republic of China: Some Keys to Success’, *Management International Review*, 28, 1 (1988), 5–6.

22 C. Blackman, *China business: the rules of the game* (St Leonards 2000), 10.

23 Tscharner, ‘China als Markt für die schweizerische Exportindustrie’, 15 May 1979, AfZ IBVA 380.3.7.

24 ‘Note interne’, 14 June 1978, AfZ IBVA 380.3.9; ‘Les relations économiques bilatérales’, August 1978, AfZ IBVA 380.3.9; Swiss Society of Chemical Industries, ‘Aide-mémoire’, undated, AfZ IBVA 380.3.9; Erb, ‘Memorandum’, 19 August 1976, IVBA 380.3.9; Hutzli, ‘Die wirtschaftlichen Beziehungen zwischen der Schweiz und der Volksrepublik China’, 1978, AfZ IBVA 380.3.9.

25 R. Konrad, *Chancen und Risiken von Equity Joint Ventures in der Volksrpublik China* (St. Gallen 1989), 113–4.

26 Imhoof, interview.

27 Zheng, *Zhonghua Renmin Gongheguo,* 141; ‘Issues on China's Utilization of Foreign Capital’, in Policy Research Department and Foreign Investment Administration, *Guide to China's Foreign Economic Relations*, 59–162; M. M. Pearson, ‘The Erosion of Controls over Foreign Capital in China, 1979-1988: Having Their Cake and Eating It Too?’, *Modern China*, 17, 1 (1991), 112–6; F. C. Rich, ‘Joint Ventures in China: The Legal Challenge’, *The International Lawyer*, 15, 2 (1981), 185–6; Ho and Huenemann, *China's Open Door Policy*, 2 and 73–79; K. Haerri, interview with A. Knüsel, 20 October 2017.

28 Li, *Breaking Through*, 241–7.

29 Jolles, ‘Bericht über China-Mission (18. bis 29. September 1978)’, 5 October 1978, AfZ NL Paul R. Jolles 1127; ‘Die erstaunlichen Chinesen’, 29 November 1978, AfZ NL Paul R. Jolles 1127; Jolles to Swiss Embassy in Beijing, 27 November 1978, AfZ NL Paul R. Jolles 1127.

30 U. Sigg, interview with A. Knüsel, 7 February 2018 (quote); Haerri, interview; Ho and Huenemann, *China's Open Door Policy*, 90-1; Zheng, *Zhonghua Renmin Gongheguo*, 142; H. Truoel, *China Schindler: My Consulting Missions*, unpublished memoir, 2002.

31 Veyrassat and Rühl, ‘Protokoll der 7. Sitzung des Vororts im Vereinsjahr 1979/1980 vom 28.1.1980’, undated, AfZ IBVA 1.5.3.7 (quote); D. Dreyer, correspondence with A. Knüsel, 13 April 2019; G. von Salis, interview with A. Knüsel, 19 January 2018.

32 G. Melancon, ‘Honour in Opium? The British Declaration of War on China, 1839–1840’, *The International History Review*, 21, 4 (1999), 855-74; A. Shai, *The Fate of British and French Firms in China, 1949-54: Imperialism Imprisoned* (Basingstoke 1996); Albers, *Britain*, 77–9.

33 ‘Laws and Regulations Concerning External Economic Relations’, in Policy Research Department and Foreign Investment Administration, *Guide*, 187(quote)-189. See also: S.-M. Shen, ‘China's New Law on Joint Ventures Using Chinese and Foreign Investment’, *Southwestern Law Journal*, 34, 5 (1981), 1183–97.

34 ‘The Agreement on the Establishment of a Joint Venture Elevator Company in the People's Republic of China’, undated, AfZ IBVA 380.3.14; ‘Articles of Association of China-Schindler Elevator Col, LTd. JVC’, undated, AfZ IBVA 380.3.14; Sigg, interview.

35 Britt, ‘Report on “Joint Venture” Discussions in Beijing-November 1979’, 20 December 1979, Novartis Archive (NOA) FCG PH 8.06.02; ‘Joint Venture Jing-Rui Pharmaceutical Company LTD. Feasibility Study Report’, October 1985, NOA FCG PH 8.06.02; ‘Minutes covering the Meeting No. 3/88’, 17 May 1988, NOA FCG RD 9.1.10.

36 ‘Chine’, 19 December 1978, NA CH AHN NES C.1.5/4250.

37 Liu, *Xin Zhongguo,* 119.

38 Gewirtz, *Unlikely Partners*.

39 Weibel, ‘Besuch von Herren der Chinesischen Botschaft’, 1 March 1979, AfZ IBVA 380.3.9.

40 Sigg, interview. See also Ho and Huenemann, *China's Open Door Policy*, 75 and 80; Jiang and Zhang, *Zhongguo*, 330, 336, 682; Zheng, *Zhonghua Renmin Gongheguo,* 164.

41 Sigg interview; Konrad, *Chancen*, 119–­20.

42 Horat and Mermod to Vorort, 12 August 1987, AfZ IBVA 380.6.3; ‘China kauft die Hühnerfarm in der Schweiz’, *Berner Zeitung* (17 June 1980).

43 Blackman, *China business* , 30–1.

44 Konrad, *Chancen*, 114.

45 C. Blocher, interview with A. Knüsel and R. Weber, 6 September 2023.

46 ‘Unsterblicher Mythos vom Riesenmarkt’, *Schweizerische Handelszeitung* (11 June 1981, quote); Zheng, *Zhonghua Renmin Gongheguo*, 190–1.

47 M. Müller, ‘Internationale Verflechtung’, in P. Halbeisen, M. Müller, B. Veyrassat (eds), *Wirtschaftsgeschichte der Schweiz im 20. Jahrhundert* (Basel 2012), 413–4 and 464–5.

48 Grisard to Schurtenberger, 1 September 1988, SFA E7115A#1996/317#1707*; Müller to EVD BIGA, 11 February 1985, SFA E2200.174#1998/182#21*; Schurtenberger, correspondence with A. Knüsel, 2 April 2019; Siber Hegner Holding AG, *Jahresbericht zum Jubiläum* (1989/1990), Schweizerisches Wirtschaftsarchiv (SWA) Banken 2004; Werner, ‘China: Besprechung vom 29. Oktober 1979’, 31 October 1979, NOA Sandoz C 951.002; Regionsdienste Region 7, ‘Volksrepublik China – Statusbericht’, 7 February 1980, NOA FCG RD 9.1.10.

49 Stuecklin, ‘People's Republic of China: Minutes of a Meeting held on November 29. 1978’, 1 December 1978, NOA FCG PH 8.06.02.

50 ‘CIBA-GEIGY Vertretung, Peking, Division Pharma’, 12 January 1979, NOA FCG PH 8.06.02 (quote); Poupon, ‘Volksrepublik China’, 2 May 1985, NOA FCG RD 9.1.10.

51 Blocher, interview; Imhoof, interview.

52 Blackman, *China business*, 34.

53 Blackman, *China business*, 24–39.

54 Blocher interview; P. Hutzli, interview with A. Knüsel, 28 July 2016.

55 Siber Hegner Holding AG, *Jahresbericht 1984/1985*, Zentralarchiv DK Group (ZDKG), C 1.26; Siber Hegner Holding AG, *Jahresbericht 1985/1986*, ZDKG C. 126; Siber Hegner Holding AG, *Jahresbericht 1986/1987*, ZDKG C 1.26; Verein Schweizerischer Textilindustrieller to Vorort, 6 August 1987, IBVA 380.6.3; R. Gerber, ‘Seidenhandel und –produktion: Krise und unternehmerische Künstler’, in Christian Kolbe (ed), *Seide: Stoff für Zürcher Geschichte und Geschichten* (Zürich 1999), 120–1.

56 Chevallaz and Huber, ‘14. Bericht zur Aussenwirtschaftspolitik vom 30. Januar 1980’, 30 January 1980, *Bundesblatt*, 1/10 (1980), 948–9.

57 Bösch, *Zeitenwende*, 172.

58 Blocher, interview. See also: A. Knüsel and R. Weber, *Die Schweiz und China: Von den Opiumkriegen bis zur neuen Seidenstrasse* (Zürich 2024).

59 Sulzer Frères to OSEC, 18 July 1980, AfZ IBVA 380.3.16.

60 ‘Handelskammer China-Schweiz’, 3 June 1980, AfZ IBVA 380.3.16; ‘Statuten der Handelskammer China-Schweiz’, undated, AfZ IBVA 380.3.16; Bäumli to Wagner, 11 April 1989, Stadtarchiv Zürich V.B. c.64, 3.11.2.2 box 1105; Debrunner to Schurtenberger, 14 April 1988, SFA E7115A#1996/317#1707*.

61 C. Liu, ‘Foreign Investment Regulations with Chinese Characteristics’, in: Policy Research Department, *Guide*, 172; Jiang and Zhang, *Zhongguo Jingji Fazhan Shi*, 336.

62 ‘Liste von Zusammenarbeitsprojekten Schweizerischer Firmen mit China’, 12.1988, SFA E2200.174#1998/181#58*.

63 Weibel, ‘Besuch von Herren der Chinesischen Botschaft.’, 23 January 1979, AfZ IBVA 380.3.9; Weibel, ‘Besuch bei der Handelsabteilung der Chinesischen Botschaft’, 1 March 1979, AfZ IBVA 380.3.9.

64 Albers, ‘Partners’, 700; O. Wolff von Amerongen, *Der Weg nach Osten: Vierzig Jahre Brückenbau für die deutsche Wirtschaft* (München 1992).

65 A. Knüsel, *China's European Headquarters: Switzerland and China in the Cold War* (Cambridge 2022); Albers, ‘Partners’, 700.

66 Blackman, *China business,* 80.

67 Text is underlined in the original: Swiss Embassy in Beijing, ‘Wirtschaft der VR China: joint ventures’, July 1980, AfZ IBVA 380.3.15.

68 Blocher, interview; Brown, Boveri & Cie., *Brown Boveri Geschäftsbericht 1978*, Historisches Archiv ABB (HA ABB) B.0.1.1.1.80; Brown, Boveri & Cie., *Brown Boveri Geschäftsbericht 1979*, HA ABB B.0.1.1.1.81; ‘Protokoll der 315. Konzernleitungssitzung’, 3 June 1985, HA ABB 562071; A. Bálint, *Sulzer im Wandel: Innovation aus Tradition* (Baden 2015), 113–4.

69 ‘Liste von Zusammenarbeitsprojekten Schweizerischer Firmen mit China’, 12.1988, SFA E2200.174#1998/181#58*. For Germany see Bösch, *Zeitenwende*, 177.

70 Swiss Embassy in Beijing, ‘Volksrepublik China’, 1 December 1979, AfZ IBVA 380.3.15; ‘3. Sitzung der gemischten Kommission Schweiz-China Oktober 1979 in Peking’, undated, AfZ IBVA 380.3.15; Schurtenberger, ‘Protokollnotiz’, 29 October 1979, AfZ IBVA 380.3.15; ‘Protokoll der 16. Direktions-Sitzung’, 18 January 1979, HA ABB 606230; Veyrassat and Hutzli, ‘Chine’, 4 January 1980, AfZ IBVA 380.3.15; ‘Protokoll der 16. Direktions-Sitzung’, 25 September 1980, HA ABB 553529. See also Taube, ‘Economic Relations’, 83.

71 ‘Les relations Suisse-Chine’, October 1986, SFA E2200.174#1998/182#22*; Wang to Schurtenberger, 21 November 1988, SFA E2200.174#1998/182#41*; Bundesamt für Aussenwirtschaft, ‘Die schweizerisch-chinesischen Wirtschaftsbeziehungen’, 20 March 1985, SFA E7115A#1993/340#1926*; Ren Z., ‘ZhongRui guanxi buduan miqie- Ruishi guomin yuan yizhang da jizhe wen’, *Liaowang Zhoukan* 42 (1984), 6; Peter Achten, ‘Schlechtwetter-Abkommen mit China’, *Tages-Anzeiger* (13 November 1986).

72 Jiang and Zhang, *Zhongguo*, 701; Dreyer, ‘China: Durch OECD-Länder gewährte Kredite zu Vorzugsbedingungen’, 25 February 1986, SFA E2010-01A#1994/372#133*; U.S. Congress, Office of Technology Assessment (ed), *Technology Transfer to China: OTA-ISC-230* (Washington, D.C. 1987), 140–2; ‘Protokoll der 298. CH-Sitzung’, 22 August 1985, HA ABB 553528.

73 Pini, *Italia e Cina*, 190-2; Samarani and Deo Giorgi, *Lontante Vicine*, 140–1.

74 B. von Tscharner, *Aussenwirtschaft und Aussenwirtschaftspolitik der Schweiz* (Zürich 1984), 77–81; Jolles, ‘Die Schweiz in den Bestrebungen nach Neuordnung’, 74–6; Presse- und Informationsdienst, ‘Abkommen über einen Mischkredit mit China’, undated [1984], SFA E7115A#1993/340#1928*; ‘Memorandum of understanding”, 23 September 1987, SFA E7115A#1996/317#1691*; ‘Liste von Zusammenarbeitsprojekten Schweizerischer Firmen mit China’, December 1988, SFA E2200.174#1998/181#58*; Bundesamt für Aussenwirtschaft, ‘Die schweizerisch-chinesischen Wirtschaftsbeziehungen’, 20 March 1985, SFA E7115A#1993/340#1928*.

75 ‘Schweizer Exportkredit für Kraftwerk in China’, *NZZ* (22 December 1987); ‘China erhält Schweizer Exportkredit’, *Tages-Anzeiger* (22 December 1987); ‘Protokoll der 294- CH-Sitzung’, 27 June 1985, HA ABB 553528.

76 Arioli, ‘9. Tagung der GK Schweiz-China’, 19 January 1988, SFA E7115A#1996/317#1691*; M. Widmer (ed), *Sieben X Seide: Die Zürcher Seidenindustrie 1954-2003* (Baden 2004), 135 and 139–41. See also Schweizerischer Exportverband: Garne, Gewebe, Stickereien to Hutzli, 6 August 1987, IBVA 380.6.3.

77 Honegger and Nef to Gebrüder Sulzer, 29 January 1985, SFA E7115A#1993/340#1904*; Blocher, interview.

78 Liu, *Xin Zhongguo,* 101–7 and 137; Zheng, *Zhonghua Renmin Gongheguo,* 133–136; Jiang and Zhang, *Zhongguo*, 329–330.

79 ‘Schweizer Manager in China harren aus’, 21 November 1986, *Tages-Anzeiger*; N. R. Lardy, ‘Chinese Foreign Trade’, in R. F. Ash and Y. Y. Kueh (eds), *The Chinese Economy under Deng Xiaoping* (Oxford 1996), 218; Ho and Huenemann, *China's Open Door Policy*, 100–1; ‘Introduction to some joint venture, join co-production & compensation trade items’, in Policy Research Department, *Guide*, 279; P. Hutzli, interview.

80 R. J. Semenik, N. Zhou and W. L. Moore, ‘Chinese Managers’ Attitudes toward Advertising’, *Journal of Advertising*, 15, 4 (1986), 56–62 (quote); Mantzopoulos and Shen, *The Political Economy*, 15–6 and 42–3; A. Hu, ‘The Distinctive Transition of China's Five-Year Plans’, *Modern China*, 39, 6 (2013), 629–39.

81 ‘Minutes covering the Meeting No. 3/88’, 17 May 1988, NOA FCG RD 9.1.10; Sigg, interview; Ho and Huenemann, *China's Open Door Policy*, 97 and 100.

82 Sigg, interview; Ho and Huenemann, *China's Open Door Policy*, 62, 93–95 and 99 (quote); ‘Introduction to some joint venture, join co-production & compensation trade items’, in: Policy Research Department, *Guide*, 279; E. T. de Bruijn and X. Jia, ‘Managing Sino-Western Joint Ventures: Product Selection Strategy’, *Management International Review* 33, 4 (1993), 343.

83 Sigg, interview. See also Truoel, *China Schindler*, 89.

84 A. Knüsel, ‘“The tea merchant has returned”: The intelligence activities of the Chinese Consulate-General in Geneva’, in: Sue Onslow and Lori Maguire (eds.), *Consuls and Consulates in the Cold War* (Leiden 2023), 82–107; A. Joske, *Spies and Lies: How China's Greatest Covert Operations Fooled the World* (Richmond 2022); Knüsel, *China's European Headquarters*; W. C. Hannas and D. K. Tatlow (eds), *China's Quest for Foreign Technology: Beyond Espionage* (Abingdon, 2021); P. Mattis and M. Brazil, *Chinese Communist Espionage: An Intelligence Primer* (Annapolis 2019); X. Guo, *China's Security State: Philosophy, Evolution, and Politics* (New York 2014); W. C. Hannas, J. Mulvenon and A. B. Puglisi, *Chinese Industrial Espionage: Technology acquisition and military modernization* (Abingdon and New York 2013); P. L. Mattis, ‘Assessing Western Perspectives on Chinese Intelligence’, *International Journal of Intelligence and CounterIntelligence*, 25, 4 (2012), 678–99; N. Eftimiades, *Chinese Intelligence Operations* (1994).

85 Shanghai Shi Renmin Zhengfu Ban to Gong Ting Tou Shi Jin Chukou Bangongshi, 4 November 1981, SMA B1-9-491-62; Shanghai Shi Fangzhi Gongye Ju to Shi Jin Chukou Ban, 15 October 1981, SMA B1-9-491-62; Siber Hegner & Co. Ltd. to Shanghai Sewing Thread and Narrow Fabric Industrial Corp., 27 August 1981, SMA B1-9-491-62; Imhoof, interview; Knüsel, *China's European Headquarters*.

86 ‘Protokoll der 11. Direktions-Sitzung’, 3 July 1980, HA ABB 553529.

87 Blocher, interview.

88 Keller to Informationsdienst, 28 January 1965, SFA E2001E#1978/84#2384*; Bernoulli to Bühler, 8 September 1958, dodis.ch/15257.

89 Meier, ‘Kommerzielle Verhandlungen mit der Volksrepublik China: Einige Gedanken’, 29 January 1988, E7115A#1996/317#1690*; Schurtenberger to EVD Bundesamt für Aussenwirtschaft, 18 June 1980, SFA E7115A#1990/142#2312* (quote).

90 ‘Protokoll der 5. Direktions-Sitzung’, 27 March 1980, HA ABB 553529; ‘Protokoll der 11. Direktions-Sitzung’, 3 July 1980, HA ABB 553529; Federli and Lehnherr to Vorort, 14 August 1987, AfZ IBVA 380.6.3.

91 ‘Chinesen zur Ausbildung bei +GF+’, *GF intern* 14 (1980), Konzernarchiv der Georg Fischer AG 9/3.14, 4–5; ‘Protokoll der 11. Direktions-Sitzung’, 3 July 1980, HA ABB 553529; Nikitin to Braun, 2 April 1981, NOA, Firmenarchiv CIBA-GEIGY PH 8.06.02; Truoel, *China Schindler*, 43–4 and 93–4.

92 K. Gerth, *Unending Capitalism: How Consumerism negated China's Communist Revolution* (Cambridge 2020). For the popularity of Swiss watches see: Knüsel, *China's European Headquarters.*

93 H. Li, *Advertising and Consumer Culture in China* (Cambridge 2016).

94 Difang guo ying Shanghai shoubiao chang to Shanghai shi qinggongye ju, 18 July 1964, SMA B163-2-1757; Waimao bu to Zhongguo zhu Ruishi dashiguan, 3 March 1964, SMA B163-2-1757; Keller, ‘Besuch des Uhrenfabrikanten Aristide Racine’, 2 June 1964, SFA E7110#1975/31#2477*; Spycher, ‘Besucher der chinesischen Handelsabteilung’, 24 October 1966, SFA E4320C#1994/78#777*.

95 ‘Shanghai: Inauguration of an OMEGA center’, Europa Star Europe, 124 (1980); ‘LONGINES Inaugurates Watch Centre in China’, Europa Star Europe, 124 (1980).

96 J. Wegelin, ‘Fast wie an der Börse’, *Schweizerische Handelszeitung* (15 June 1989 quote); ‘Schweizer Uhrenzentrum in China eröffnet’, *NZZ* (7 October 1981); Chambre Suisse de l’Horlogerie, *Rapport 1981* (1982), 17, SWA Bh 13.

97 Konrad, *Chancen*, 118; Y. Wang, ‘Intention to stay in Chinese foreign-invested companies: previous experiences, initial expectations and current satisfaction’, *The International Journal of Human Resource Management*, 21, 11 (2010), 1996–2012.

98 Arioli, ‘9. Tagung der GK Schweiz-China’, 19 January 1988, SFA E7115A#1996/317#1691*; Widmer, *Sieben X Seide*, 135 and 139–41.

99 Nikitin to Braun, 2 April 1981, NOA FCG PH 8.06.02; Dürler and Nikitin to Ma, 16 July 1981, NOA FCG PH 8.06.02; Swiss Society of Chemical Industries, ‘Aide mémoire’, 25 January 1987, SFA E7115A#1996/317#1707*; Dreyer to Liu, 16 March 1988, SFA E7115A#1996/317#1707*.

100 Jacobs Suchard AG to Meier, 3 December 1987, SFA E2200.174#1998/182#18*; Swiss Embassy in Beijing to Ou, 14 December 1987, SFA E2200.174#1998/182#18*; Jacobs Suchard AG to Meier, 7 September 1987, SFA E2200.174#1998/182#18*; Dreyer to Jacobs Suchard AG, 5 February 1987, SFA E2200.174#1998/182#18*; Jacobs Suchard AG to Meier, 16 June 1986, SFA E2200.174#1998/182#18*; Jacobs Suchard AG to Meier, 28 May 1986, SFA E2200.174#1998/182#18*.

101 Kraus, ‘More than Just a Soft Drink,’ 116–8.

